# Sufentanil and Bupivacaine Combination versus Bupivacaine Alone for Spinal Anesthesia during Cesarean Delivery: A Meta-Analysis of Randomized Trials

**DOI:** 10.1371/journal.pone.0152605

**Published:** 2016-03-31

**Authors:** Jiajia Hu, Chengliang Zhang, Jianqin Yan, Ruike Wang, Ying Wang, Mu Xu

**Affiliations:** 1 Department of Anesthesiology, Xiangya Hospital, Central South University, Changsha, China; 2 Department of Cardiovascular Surgery, Xiangya Hospital, Central South University, Changsha, China; 3 Department of Anesthesiology, Fujian Provincial Cancer Hospital, Teaching hospital of Fujian Medical University, Fuzhou, China; University of Bari, ITALY

## Abstract

**Objective:**

The addition of lipophilic opioids to local anesthetics for spinal anesthesia has become a widely used strategy for cesarean anesthesia. A meta-analysis to quantify the benefits and risks of combining sufentanil with bupivacaine for patients undergoing cesarean delivery was conducted.

**Methods:**

A comprehensive literature search without language or date limitation was performed to identify clinical trials that compared the addition of sufentanil to bupivacaine with bupivacaine alone for spinal anesthesia in healthy parturients choosing cesarean delivery. The Q and I^2^ tests were used to assess heterogeneity of the data. Data from each trial were combined using relative ratios (RRs) for dichotomous data or weighted mean differences (WMDs) for continuous data and corresponding 95% confidence intervals (95% CIs) for each trial. Sensitivity analysis was conducted by removing one study a time to assess the quality and consistency of the results. Begg’s funnel plots and Egger’s linear regression test were used to detect any publication bias.

**Results:**

This study included 9 trials containing 578 patients in the final meta-analysis. Sufentanil addition provided a better analgesia quality with less breakthrough pain during surgery than bupivacaine alone (RR = 0.10, 95% CI 0.06 to 0.18, P < 0.001). Sensory block onset time was shorter and first analgesic request time was longer in sufentanil added group compared with the bupivacaine-alone group (WMD = −1.0 min, 95% CI −1.5 to −0.58, P < 0.001 and WMD = 133 min, 95% CI 75 to 213, P < 192, respectively). There was no significant difference in the risk of hypotension and vomiting between these two groups. But pruritus was more frequentely reported in the group with sufentanil added (RR = 7.63, 95% CI 3.85 to 15.12, P < 0.001).

**Conclusion:**

Bupivacaine and sufentanil combination is superior to that of bupivacaine alone for spinal anesthesia for cesarean delivery in analgesia quality. Women receiving the combined two drugs had less breakthrough pain, shorter sensory block onset time, and longer first analgesic request time. However, the addition of sufentanil to bupivacaine increased the incidence of pruritus.

## Introduction

It was suggested adding opioids to local anesthetic agents for spinal anesthesia might improve anesthesia quality and prolongs the duration of action[1, 2]. Intrathecal administration of opioids is commonly used for cesarean delivery. However, the benefits and risks of this practice with opioids added remain to be fully examined and confirmed[3]. Sufentanil, a lipophilic opioid, was the most frequent drug used in conjunction with the local anesthesitic bupivacaine for cesarean delivery. The aim of this study was to review the analgesic efficacy and side effects of the addition of sufentanil to bupivacaine for spinal anesthesia in healthy parturients undergoing cesarean delivery by a meta-analysis.

## Methods

### Ethics

No ethics approval was required.

### Protocol

Meta-analysis was conducted in accordance with the reporting recommendations of the PRISMA statement and Cochrane Collaboration for systematic reviews and meta-analysis[4–6] (data in S1 Text).

### Systematic search

Full articles reporting randomized controlled trials that compared the addition of sufentanil to bupivacaine with bupivacaine alone for cesarean delivery were searched. High-sensitivity and low-specificity search principles were used in MEDLINE, Embase, Cochrane Central Register of Controlled Trials (CENTRAL), and Web of Science without any language or date limitation. The keywords “cesarean delivery”, “sufentanil”, “spinal anesthesia”, “randomized controlled trial”, and their alternative words were combined by the Boolean meanings of “AND” (for “cesarean delivery”, “sufentanil”, “spinal anesthesia”, “randomized controlled trial”) and “OR” (among alternative words). We also searched the reference lists of relevant articles or textbooks to find other potential studies. The last electronic search was performed in August 2015.

### Inclusion and exclusion criteria

We included published randomized controlled clinical trials that compared the addition of sufentanil to bupivacaine with identical dose bupivacaine alone used for spinal anesthesia in healthy parturients undergoing scheduled cesarean delivery. Trials that examined different bupivacaine doses between the study and control groups were excluded. Trials focused on other opioids for spinal anesthesia, or for postoperative analgesia for labour were also excluded. Trials reported in scientific meetings, correspondence, case reports, and review papers were also excluded.

### Data collection

The published papers were reviewed independently by two medical doctors (C Zhang and J Hu). Duplicate studies were excluded redundance from, and then titles, abstracts, and full texts were screened to select the trials that matched the inclusion criteria. Quality of included trials were evaluated using the Cochrane Collaboration’s tool for assessing risk of bias in randomized trials[5].

Two authors doctotors (R Wang and Y Wang) independently extracted all the relevant information from each included study. Another two doctors checked the consistencey of the extracted data. All doctors involved in data extraction had more than 5 years aneshesiology experience. For each included trial, the following data were collected: the name of the first author, publication year, number of patients, anesthetic dose, the incidence of breakthrough pain requiring supplementary systemic analgesia or conversion to general anesthesia, sensory block onset time (interval from end of anesthetic injection to loss of pain sensitivity to pinprick at predefined dermatome level), first analgesic request time (interval from end of anesthetic injection to time of postdelivery complaint of pain that required analgesia treatment), motor block onset time and duration (assessed by modified Bromage scale or Bromage scale), neonatal Apgar scores, and incidence of intraoperative maternal side effects such as hypotension (defined as percent decrease in systolic blood pressure below its baseline value or below an absoluate lower limit, and the definitions were differed among trials), nausea, vomiting, pruritus, and shivering.

The incidence of breakthrough pain, sensory block onset time, first analgesic request time, motor block onset time, and duration were analyzed as the primary outcomes.

When any there listed outcomes were not reported, the missing data were requested to the original authors. If this was unsuccessful, extrapolated data from studies were performed whenever possible[7]. In cases of conflicting evaluations, disagreements were resolved through discussions among the six doctors in the research group.

### Statistical analysis

Analysis was conducted using STATA version 12.0. Each analysis was assessed for statistical heterogeneity using the Cochran’s Q and I^2^ tests. P < 0.10, and I^2^ > 50% was considered significant. If P > 0.10, and I^2^ < 50% a fixed effects model was used for analysis; otherwise the random effects model was used. To identify sources of heterogeneity, subgroup analysis and meta-regression were conducted. Relative ratios (RRs) for dichotomous data or weighted mean differences (WMDs) for continuous data with pertinent 95% confidence intervals (95% CIs) were computed for each analyzed trial. Sensitivity analysis was conducted by removing each study individually to assess the quality and consistency of the results. Begg’s funnel plots and Egger’s linear regression test were used to detect any publication bias.

For multiple intervention groups of sufentanil, we combined intervention groups to create a single pair-wise comparison according the Cochrang handbook[8] For dichotomous outcomes, both the sample size and the number of patients with events were summed across groups. For continuous outcomes, mean and standard deviation were combined using a formula proposed by the Cochrane Collaboration[8].

## Results

### Literature search findings

**Fig 1:**
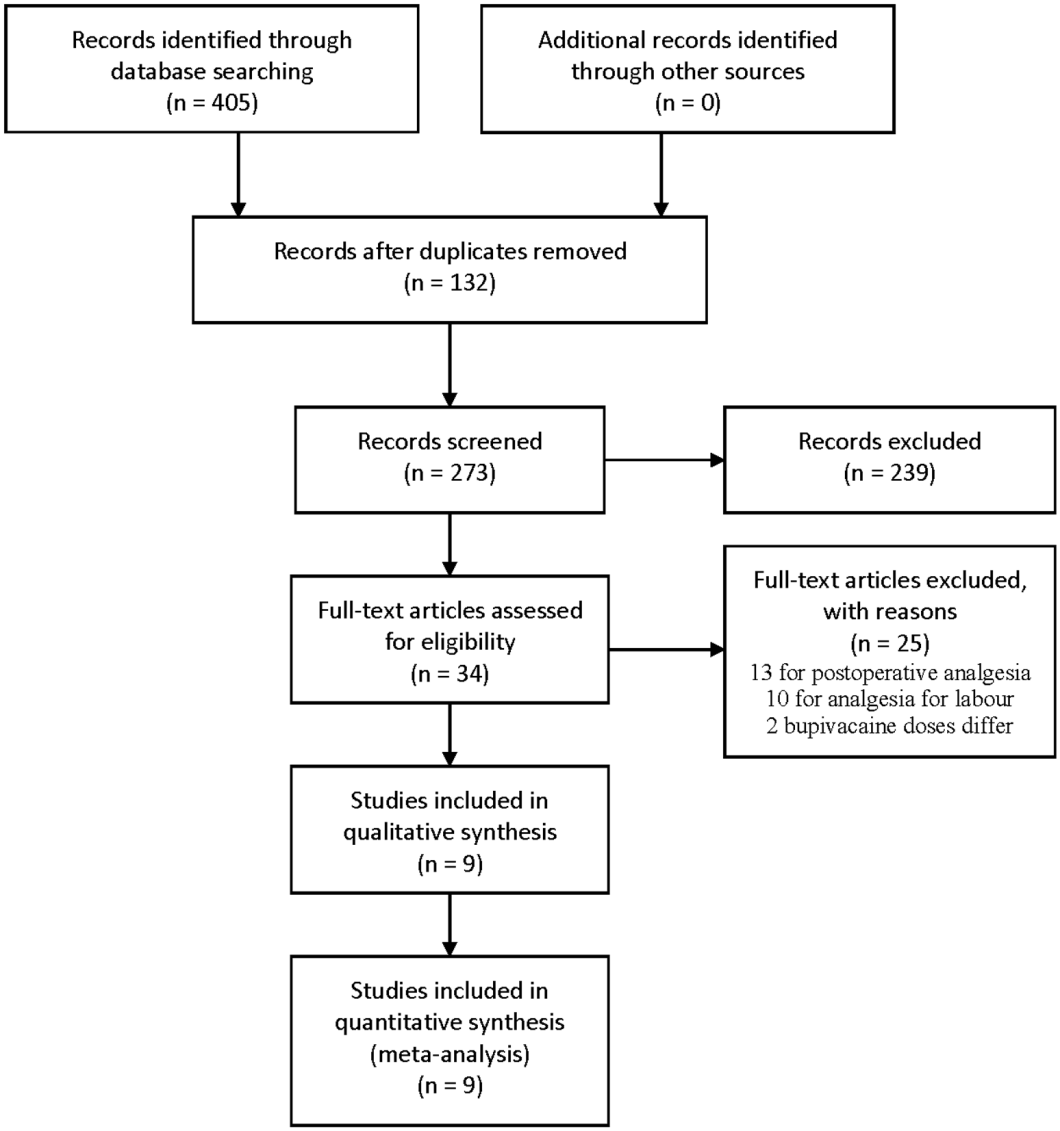
Flow chart of articles selection. The process of systematic literature search, articles retrieved, excluded, and analysed trials.

A total of 405 potentially relevant articles from our search of the literature were identified. After excluding 396 articles, a total of 9 articles covering 9 trials published between 1992 and 2012 with 578 patients included (363 received sufentanil in addition to bupivacaine). Fig 1 shows the study selection procedure. A summary of the included studies is shown in Table 1. Quality of included trials were shown in Table 2.

**Table 1:** Characteristics of included trials.

Year	First author	Bupivacaine Dose (mg)/	Sufentanil Dose (ug)	Number of Study/Control
1997	Dahlgren^[15]^	12.5 (hyperbaric)	2.5/5	40/20
1998	Ngiam^[11]^	7.5 (plain)	10	20/17
2003	Braga^[17]^	12.5 (hyperbaric)	2.5/5/7.5	60/20
2006	Demiraran^[16]^	12.5 (hyperbaric)	1.5/2.5/5.0	75/25
2010	Vyas^[13]^	11 (hyperbaric)	5	30/30
2010	Veena^[12]^	12(Not mentioned)	10	20/20
2011	Lee^[10]^	near 10 (hyperbaric)	2.5	24/24
2012	Braga^[9]^	10 (hyperbaric)	5	24/24
2012	Bang^[14]^	near 10 (hyperbaric)	2.5/5	70/35

**Table 2:** Quality of included trials assessed by Risk bias tools.

Year	First author	Random sequence generation	Allocation concealment	Blinding of participants and personnel	Blinding of outcome assessment	Incomplete outcome data	Selective reporting	Other bias
1997	Dahlgren^[15]^	Unclear	Low	Low	Low	Low	Low	Unclear
1998	Ngiam^[11]^	Unclear	Low	Low	Low	Low	Low	Unclear
2003	Braga^[17]^	Low	Low	Low	Low	Low	Low	Unclear
2006	Demiraran^[16]^	Low	Unclear	Unclear	Unclear	Unclear	Unclear	Unclear
2010	Vyas^[13]^	Unclear	Low	Low	Low	Low	Low	Unclear
2010	Veena^[12]^	Low	Low	Unclear	Unclear	Unclear	Low	Unclear
2011	Lee^[10]^	Unclear	Unclear	Unclear	Unclear	Low	Low	Unclear
2012	Braga^[9]^	Low	Low	Unclear	Unclear	Unclear	Unclear	Unclear
2012	Bang^[14]^	Unclear	Unclear	High	Unclear	Unclear	Low	Unclear

High: High risk bias

Unclear: Unclear risk bias

Low: Low risk bias

Five trials examined 1 dose of the drug sufentanil[9–13], and the rest 4 examined more than 1 dose (2 trials examined 2 doses[14, 15], 2 trials examined 3 doses[16, 17]). Trials that tested more than 1 dose were combined to create a single pair-wise comparison as previously mentioned. All the meta-analysis results were shown in Table 3.

**Table 3:** Meta-Analysis results of Comparison of sufentanil and bupivacaine combination versus bupivacaine alone.

Outcome	Trials	Number		WMD/RR	95% CI	I^2^ value	Model
		Sufentanil	Control				
Breakthrough Pain	4	138	103	0.10	(0.06, 0.18)	0.0%	Fixed
Sensory block onset	4	94	91	-1.04	(-1.50, -0.58)	0.0%	Fixed
first analgesic request time	7	264	166	133	(75, 192)	98.0%	Random
Motor block duration	2	64	44	-1.04	(-1.50, -0.58)	89.0%	Random
Hypotension	6	233	145	1.19	(0.89, 1.61)	17.5%	Fixed
Nausea	7	299	171	0.79	(0.34, 1.86)	66.9%	Random
Vomiting	6	279	154	0.86	(0.24, 3.05)	57.1%	Random
Pruritus	8	339	191	7.63	(3.85, 15.12)	0.0%	Fixed
Shivering	3	114	79	0.71	(0.42, 1.17)	49.8%	Fixed

RR: relative ratio

WMD: weighted mean difference

### Anesthesia quality

**Fig 2:**
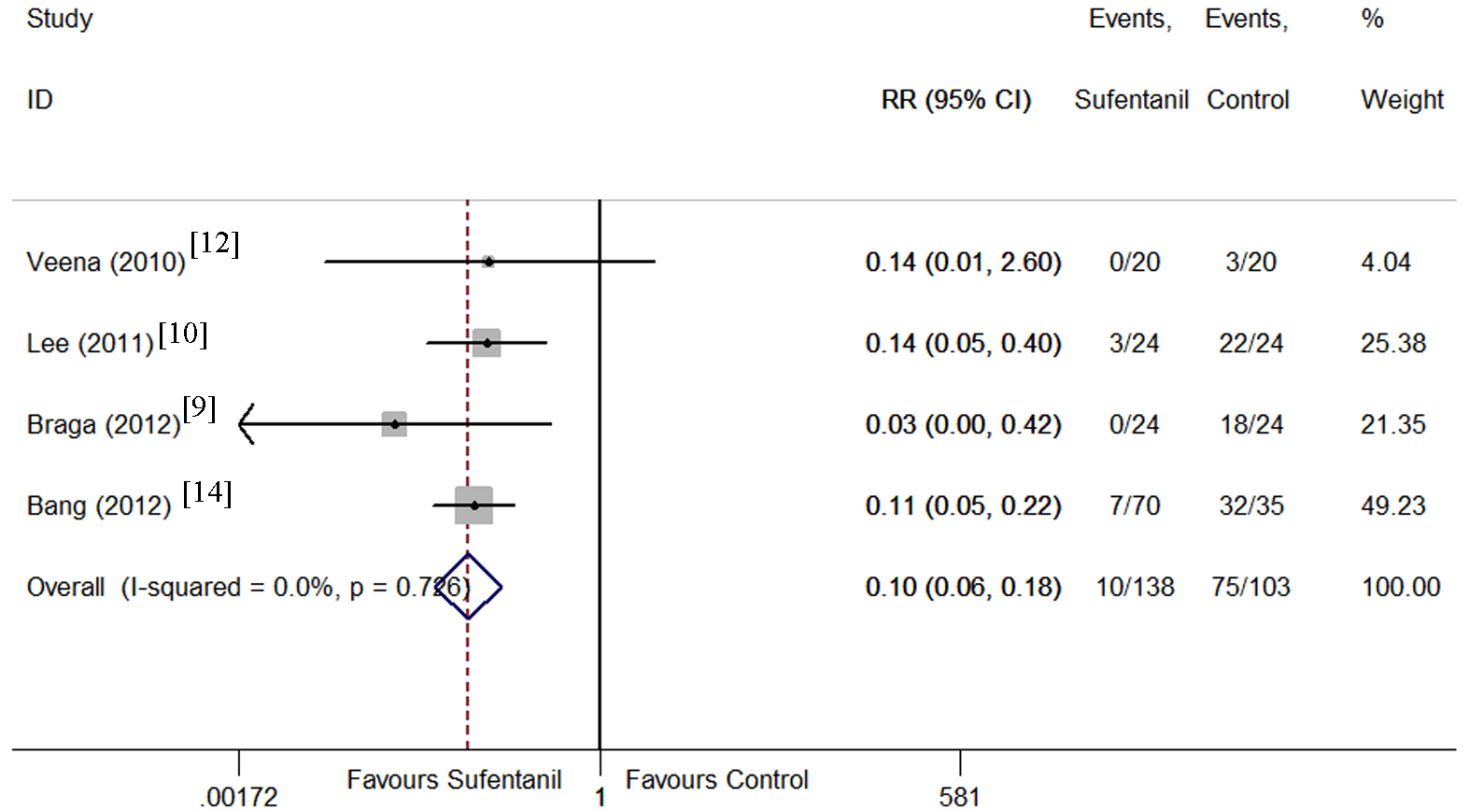
Forest plot of breakthrough pain incidence. Forest plot displaying the results of the fixed effects meta-analysis for breakthrough pain incidence in sufentanil and bupivacaine combination versus bupivacaine alone. Sufentanil, sufentanil and bupivacaine combination. Control, bupivacaine alone. RR, relative risk. CI, confidence interval.

**Fig 3:**
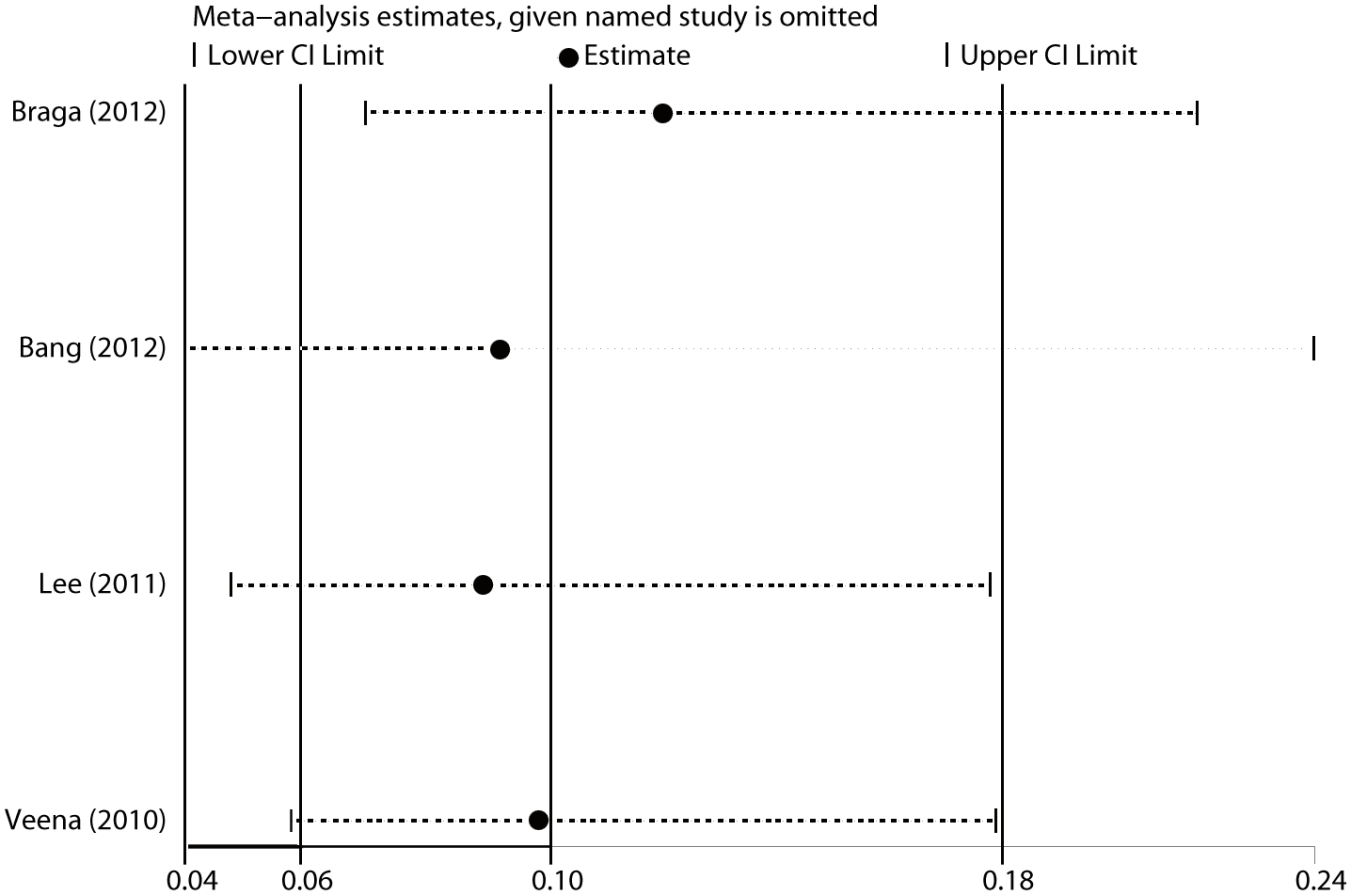
Sensitivity analysis results of breakthrough pain incidence. Sensitivity analysis was conducted by removing each study individually to assess the quality and consistency of the results. CI, confidence interval.

**Fig 4:**
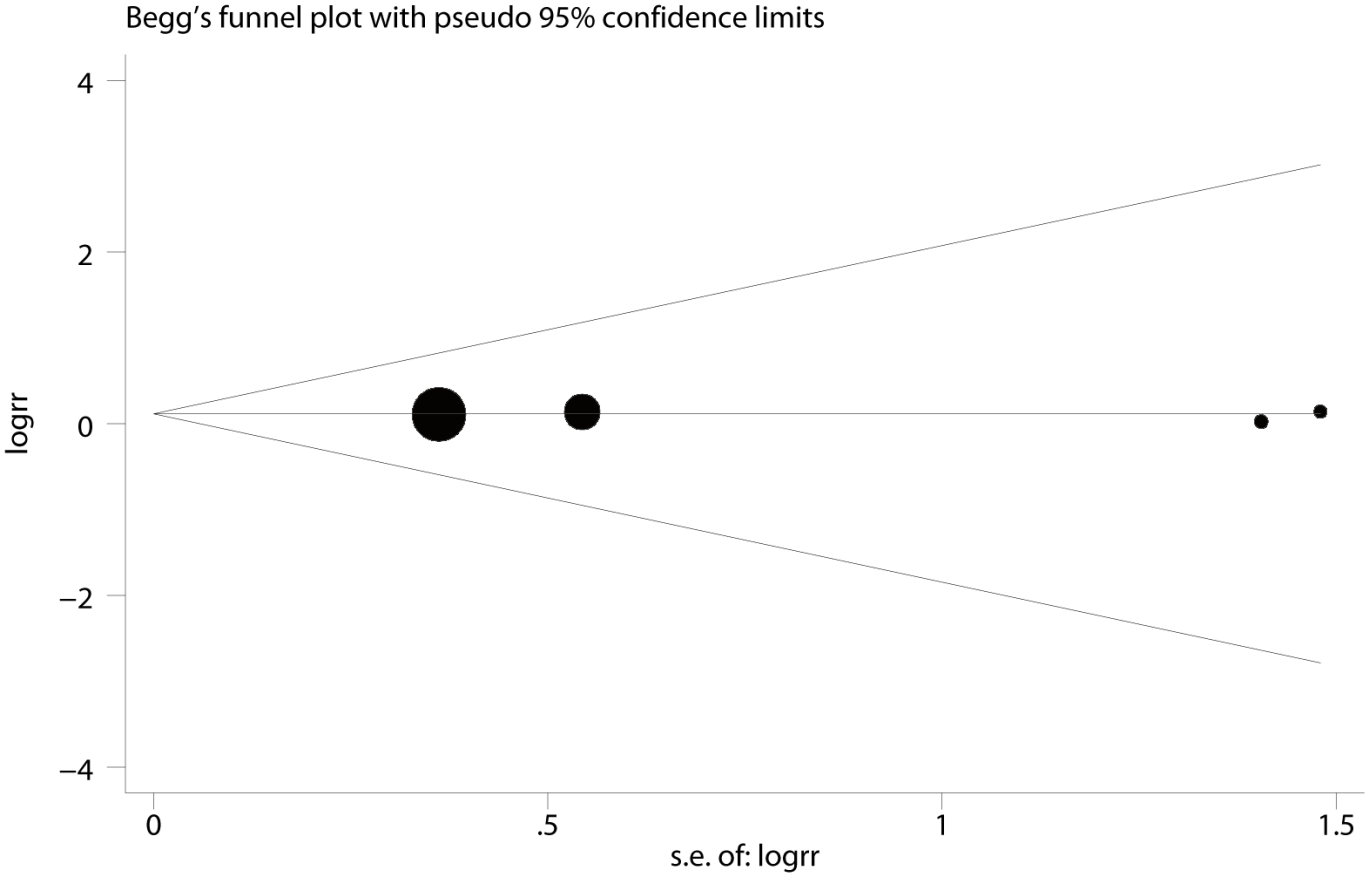
Funnel plot of breakthrough pain incidence. Funnel plot displaying the Begg’s funnel plots for breakthrough pain incidence in sufentanil and bupivacaine combination versus bupivacaine alone.

Six trials [9–12, 14, 16] were pooled using a fixed effect model since no heterogeneity was observed (I^2^ < 0.1%, P = 0.73) when their anesthesia quality were examined. Bupivacaine and sufentanil combination significantly reduced the incidence of breakthrough pain during surgery compared with bupivacaine alone(RR = 0.10, 95% CI 0.06 to 0.18 P < 0.001, Fig 2). Breakthrough pain occurred in 10 of 138 patients in sufentanil group, and in 75 of 103 in bupivacaine-alone group. The result was stable when sensitivity analysis was conducted by removing 1 trial at a time from the pooled result (RR_min_ = 0.09, 95% CI_min_ 0.04 to 0.18; RR_max_ = 0.12, 95% CI_max_ 0.07 to 0.21, Fig 3). The Begg’s funnel plots (P = 1.00) and Egger’s linear regression test (P = 0.76) indicated the probability of publication bias was low (Fig 4).

### Sensory block and motor block

**Fig 5:**
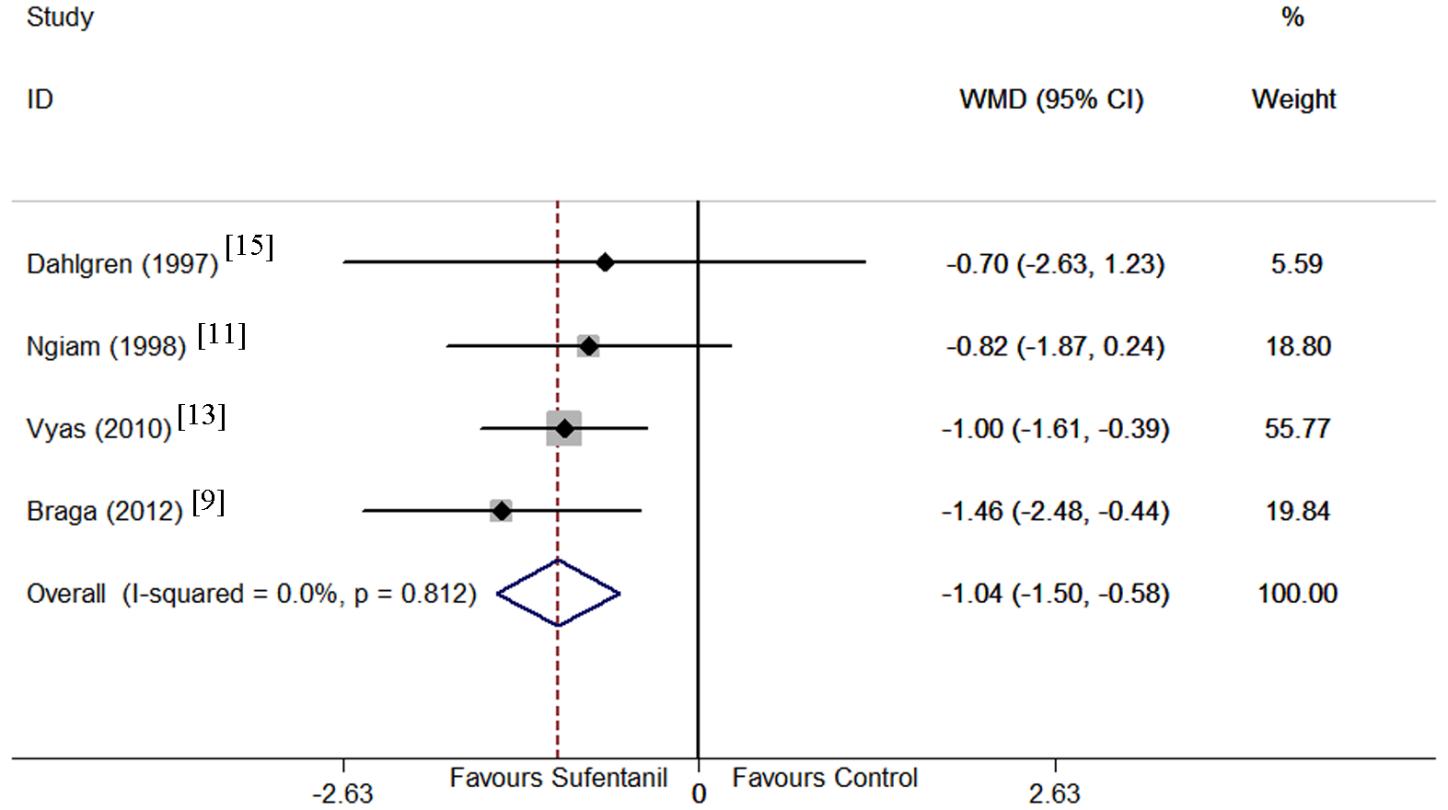
Forest plot of sensory block onset time. Forest plot displaying the results of the fixed effects meta-analysis for sensory block onset (min) in sufentanil and bupivacaine combination versus bupivacaine alone. Sufentanil, sufentanil and bupivacaine combination. Control, bupivacaine alone. WMD, weighted mean difference. CI, confidence interval.

Sensory block onset time was examined in 4 trials[9, 11, 13, 15]. No heterogeneity was observed according to the I^2^ and Q tests (I^2^ = 0.0%, P = 0.81), and therefore, the fixed effects model was selected. Pooled result suggested that sufentanil added to bupivacaine shortened sensory block onset time compared with bupivacaine alone (WMD = -1.04 min, 95% CI -1.50 to -0.58 min; P < 0.001, Fig 5). Removal of individual trials did not significantly alter the result.

**Fig 6:**
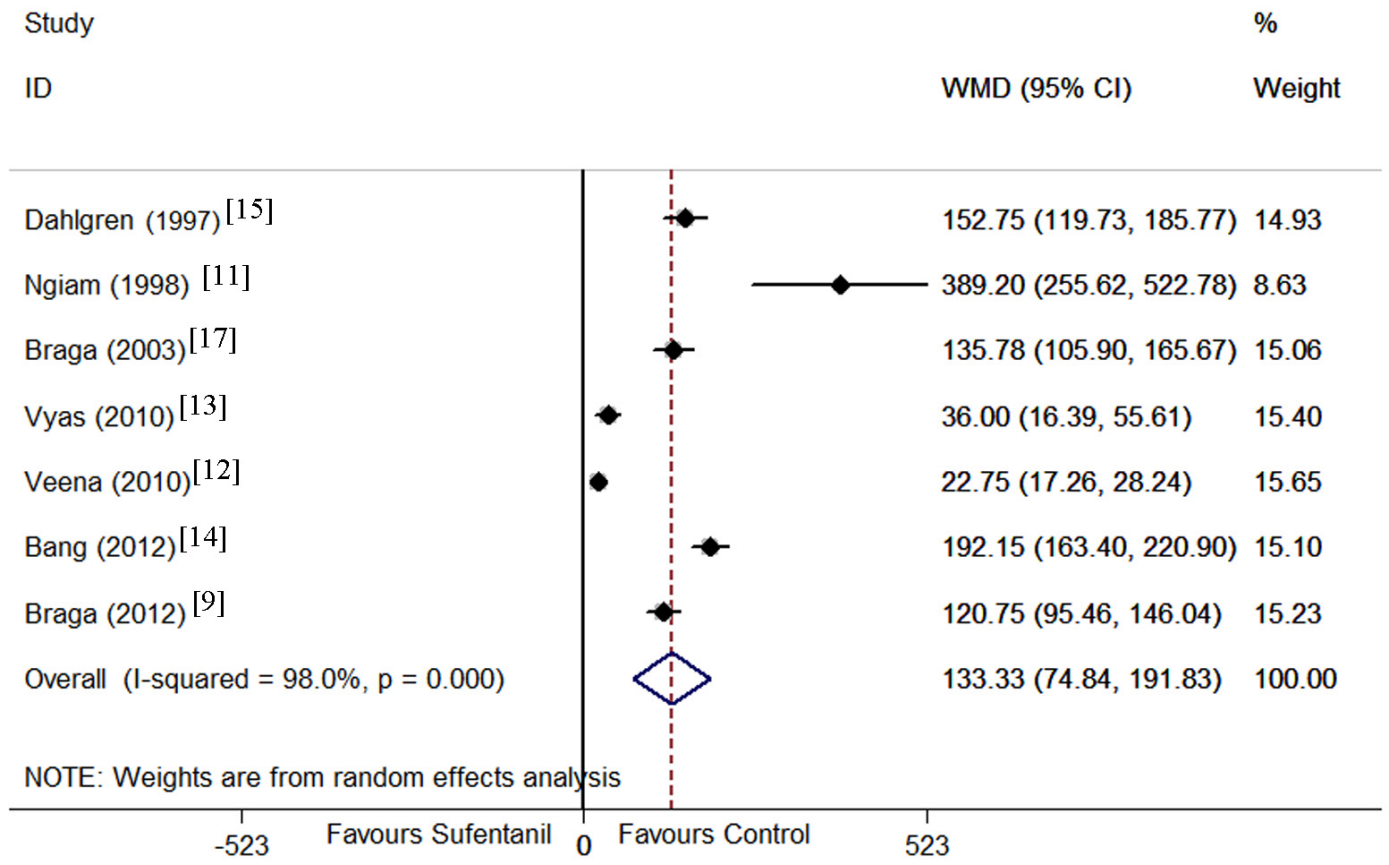
Forest plot of first analgesic request time. Forest plot displaying the results of the random effects meta-analysis forfirst analgesic request time (min) in sufentanil and bupivacaine combination versus bupivacaine alone. Sufentanil, sufentanil and bupivacaine combination. Control, bupivacaine alone. WMD, weighted mean difference. CI, confidence interval.

First analgesic request time was examined in 7 trials[9, 11–15, 17]. The possibility of heterogeneity was high (P < 0.001, I^2^ = 98.0%); therefore, subgroup analysis and meta-regression were performed to identify the sources of heterogeneity. Not any source of the heterogeneity was observed, thererfore, a random effect model was selected to pool these results, which demonstrated that the addition of sufentanil prolonged first analgesic request time compared with bupivacaine alone (WMD = 133 min, 95% CI 75 to 192, P < 0.001, Fig 6). Again these result was stable when sensitivity analysis that involved removing one trial once from the pooled result was conducted.

**Fig 7:**
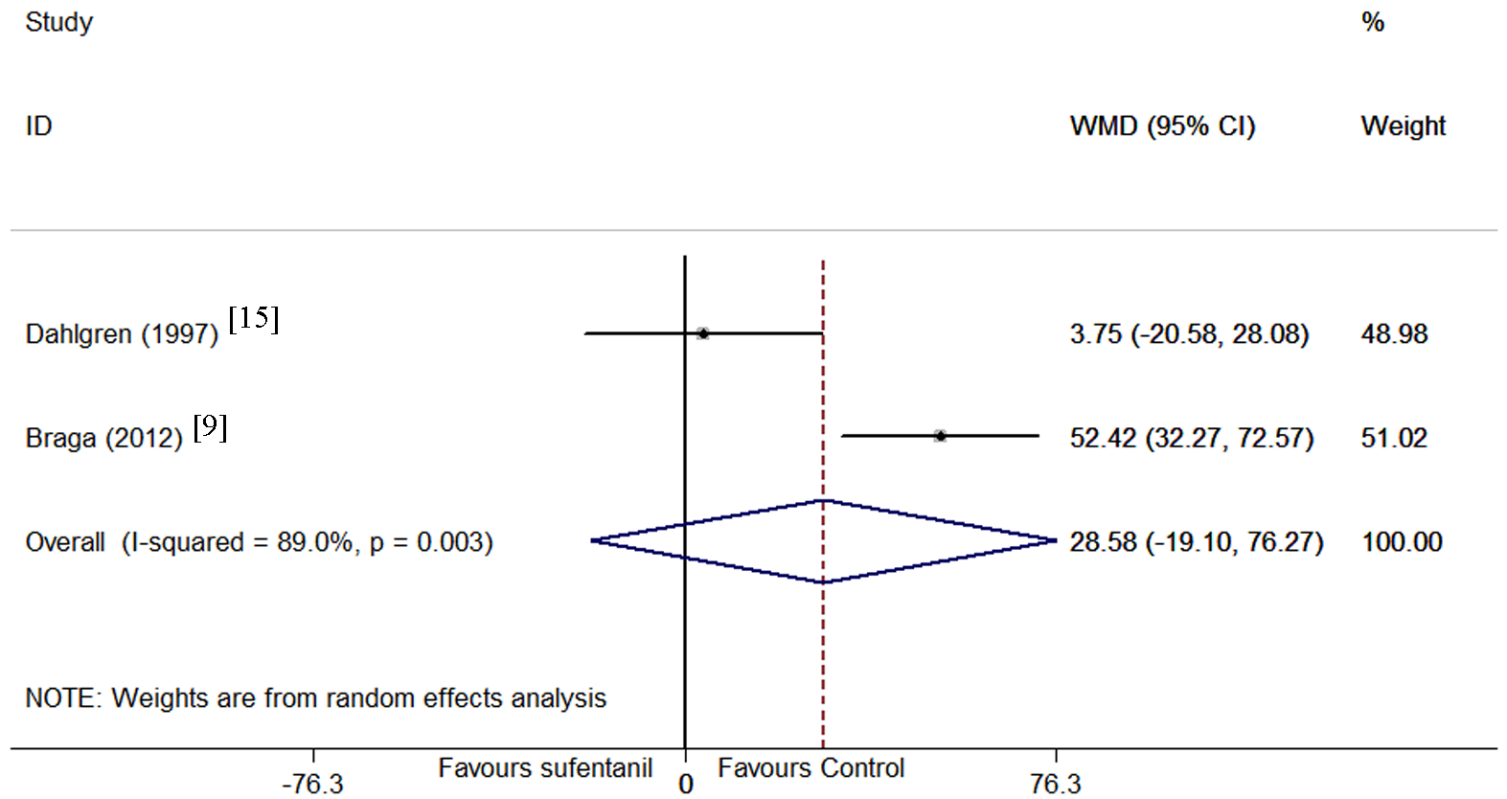
Forest plot of motor block duration. Forest plot displaying the results of the random effects meta-analysis for motor duration onset (min) in sufentanil and bupivacaine combination versus bupivacaine alone. Sufentanil, sufentanil and bupivacaine combination. Control, bupivacaine alone. WMD, weighted mean difference. CI, confidence interval.

Motor block onset time was only examined in 1 trial[15]. Motor block duration was tested in 2 trials[9, 15]. A significant heterogeneity was existed in motor block duration according to the I^2^ and Q tests (P = 0.003, I^2^ = 89.0%). Although subgroup analysis and meta-regression were performed, no source of the heterogeneity was found significant, and the results were pooled using a random effect model. The pooled results suggested that adding sufentanil to bupivacaine did not affect motor block duration compared with bupivacaine alone (WMD = 29 min, 95% CI -19 to 76, P = 0.24, Fig 7). However, when the trial of Dahlgren et al[15] was removed from the pooled trials, the 95% CI was all greater than zero.

### Neonatal data

The healthy states of the neonates were evaluated by neonatal Apgar scores 1 and 5 min after delivery. The count of Apgar scores which were lower than 7 after delivery were examined in 6 trials[9, 10, 14–17]. All the 1-min and 5-min Apgar scores were above 7 in the included trials.

### Maternal side effects

**Fig 8:**
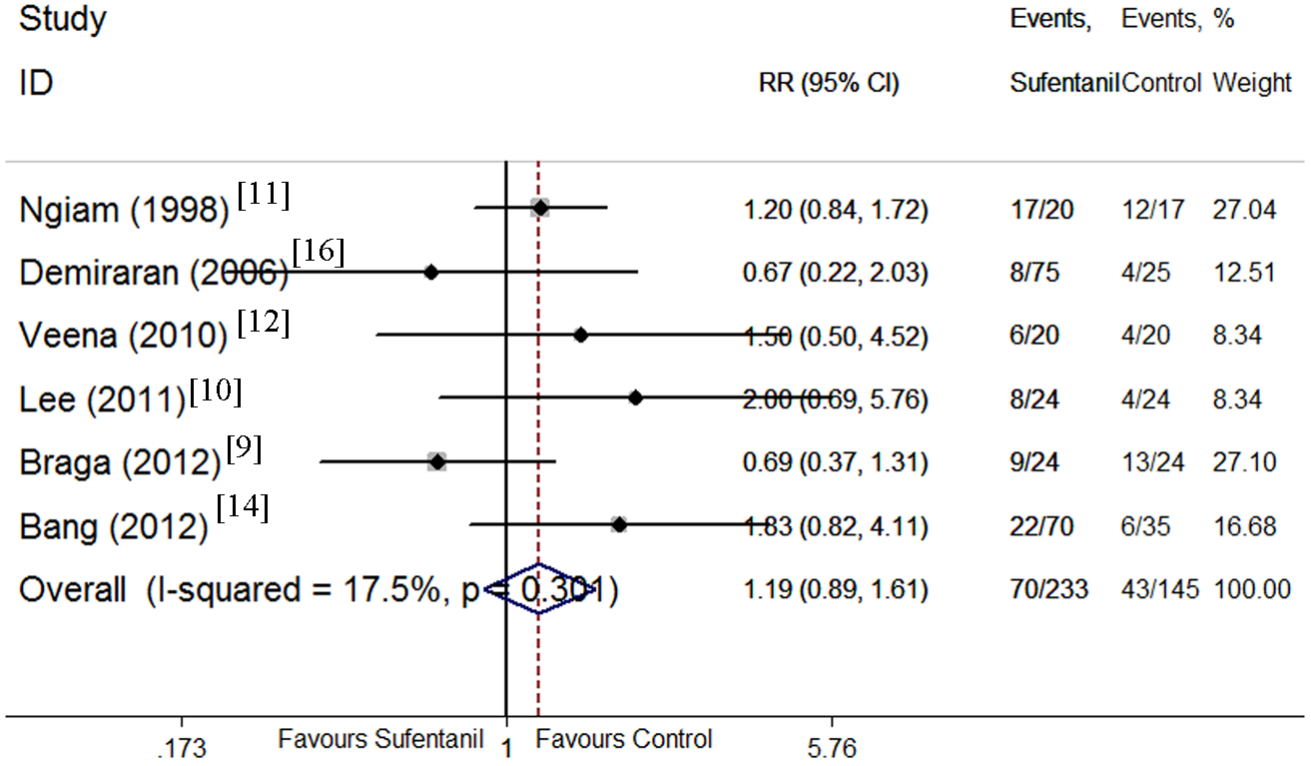
Forest plot of maternal hypotension incidence. Forest plot displaying the results of the fixed effects meta-analysis for maternal hypotension incidence in sufentanil and bupivacaine combination versus bupivacaine alone. Sufentanil, sufentanil and bupivacaine combination. Control, bupivacaine alone. RR, relative risk. CI, confidence interval.

**Fig 9:**
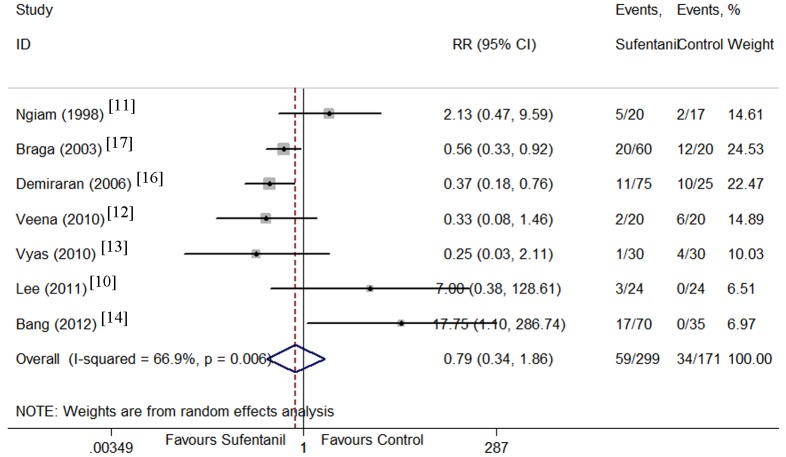
Forest plot of maternal nausea incidence. Forest plot displaying the results of the random effects meta-analysis for maternal nausea incidence in sufentanil and bupivacaine combination versus bupivacaine alone. Sufentanil, sufentanil and bupivacaine combination. Control, bupivacaine alone. RR, relative risk. CI, confidence interval.

**Fig 10:**
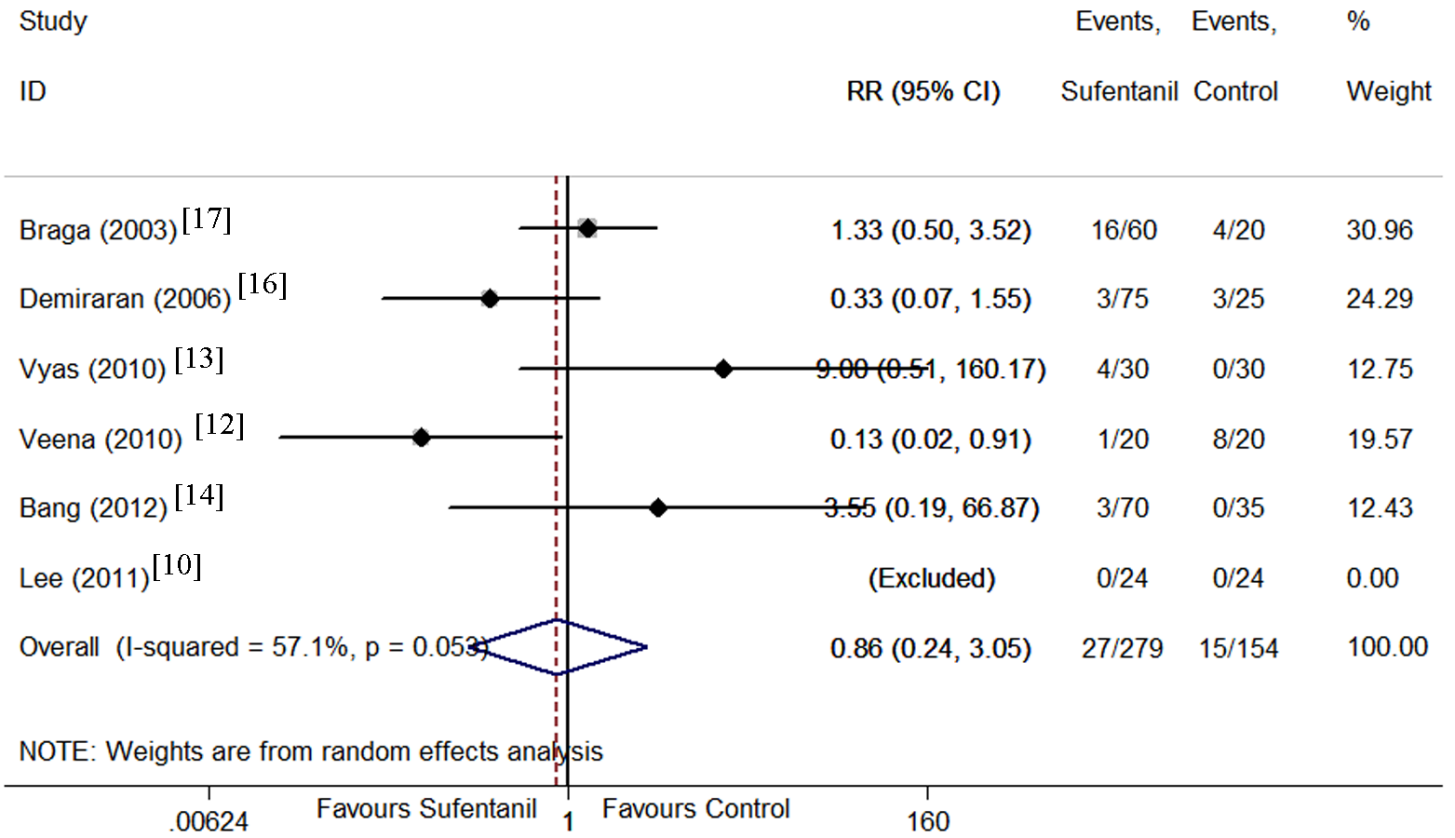
Forest plot of maternal vomiting incidence. Forest plot displaying the results of the random effects meta-analysis for maternal vomiting incidence in sufentanil and bupivacaine combination versus bupivacaine alone. Sufentanil, sufentanil and bupivacaine combination. Control, bupivacaine alone. RR, relative risk. CI, confidence interval.

**Fig 11:**
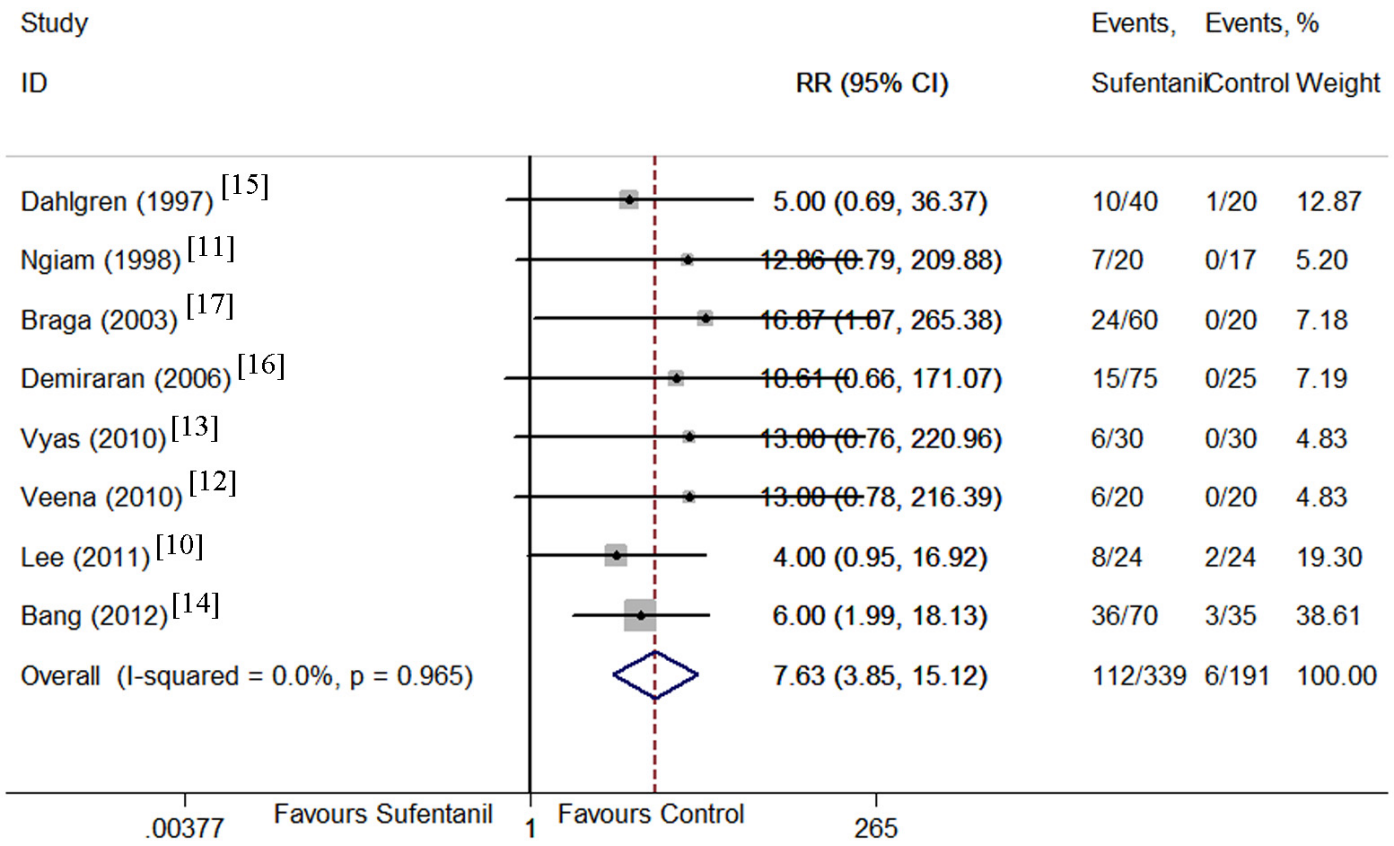
Forest plot of maternal pruritus incidence. Forest plot displaying the results of the fixed effects meta-analysis for maternal pruritus incidence in sufentanil and bupivacaine combination versus bupivacaine alone. Sufentanil, sufentanil and bupivacaine combination. Control, bupivacaine alone. RR, relative risk. CI, confidence interval.

**Fig 12:**
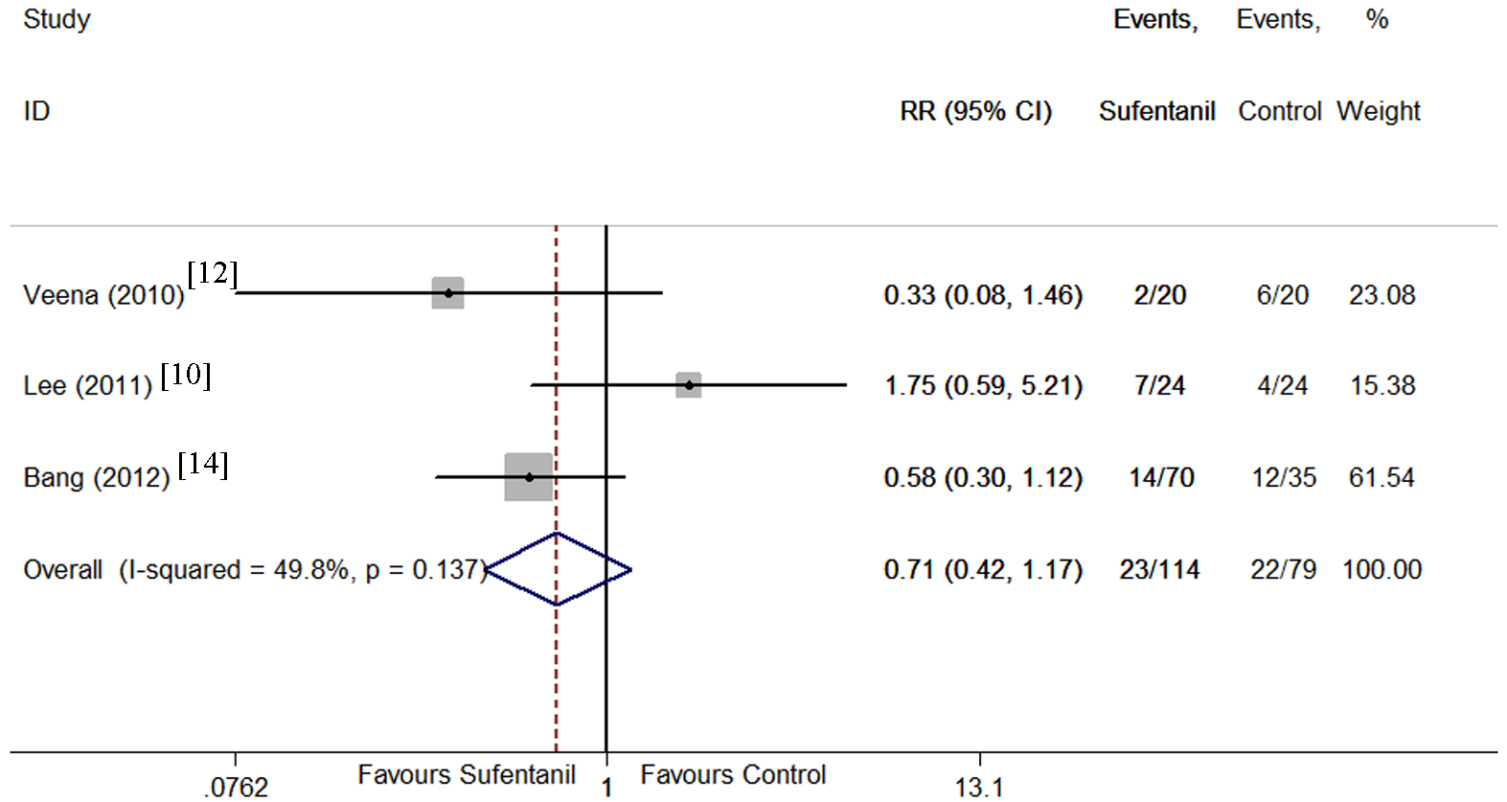
Forest plot of maternal shivering incidence. Forest plot displaying the results of the fixed effects meta-analysis for maternal shivering incidence in sufentanil and bupivacaine combination versus bupivacaine alone. Sufentanil, sufentanil and bupivacaine combination. Control, bupivacaine alone. RR, relative risk. CI, confidence interval.

Maternal side effects including hypotension [9–12, 14, 16], nausea [10–14, 16, 17], vomiting [10, 12–17], pruritus [10–18], and shivering[10, 12, 14] were compared between sufentanil added and bupivacaine alone groups. There were no significant differences in the incidence of hypotension, and vomiting. However, the CIs were wide and heterogeneity was significant. Sufentanil addition significantly increased the incidence of pruritus (Figs 8–12). Sufentanil additiondid not affect the incidence of nausea; however, when the trial of Bang et al [14] was removed from the analysis, the incidence of nausea was significantly lower in the sufentanil combined with bupivacaine group (RR = 0.58, 95% CI 0.40 to 0.85). The pooled results showed that the incidence of shivering was lower in thesufentanil added group, but this conclusion did not stand true when the trial of Lee et al [10] was removed from the analysis (RR = 0.52, 95% CI 0.28 to 0.94). Further evidences maybe required to reach a clear conclusion about the effects on the incidence of nausea and shivering of sufentanil added or bupivacaine alone.

### Additional analysis

A significant heterogeneity was identified in the analyses of first analgesic request time, motor block duration, and incidence of nausea (I^2^ > 50%, all P < 0.10). However, the sources of the heterogeneity was not found after having performed subgroup analysis and meta-regression using data sources.

Publication bias was assessed by Begg’s funnel plots and Egger’s linear regression test. All Begg’s funnel plots showed basic symmetry, and Egger’s linear regression test suggested that the probability of publication bias was low (P > 0.05).

## Discussion

Spinal anesthesia is routinely used for cesarean delivery because of its ease of control, fast onset, effective nerve block, low failure rate, and low systemic toxicity[19, 20]. In addition, it can decrease the risk of airway complications compared to general anesthesia.[20, 21]. However, using local anesthetics alone for spinal anesthesia may be inadequate[19]. It has been suggested that the addition of various opioids to local anesthetics may improve intra- and post-operative analgesic effects and reduce side effects[14, 15, 17, 20]. A previous meta-analysis had examined the effects of adding opioids for many minor surgeries but cesarean delivery was not included[2]. The present meta-analysis comparing the addition of sufentanil to bupivacaine with bupivacaine alone for spinal anesthesia found that the addition of sufentanil to bupivacaine provided significant benefits for spinal anesthesia in healthy parturients during cesarean delivery as compared to bupivacaine alone.

A clinically important effect of adding lipid-soluble sufentanil to bupivacaine is the significantly lower incidence of breakthrough pain. Studies suggested that spinal anesthesia with high-dose local anesthetic (e.g., bupivacaine 12–15 mg) provided effective analgesia, but with a high incidence of hypotension[11, 14]. Although spinal anesthesia by low-dose local anesthetic without opioid is with a low incidence of hypotension, but also a high probabilility of failed surgical anesthesia was concerned. Low-dose bupivacaine combined with opioid not only reduces the incidence of intraoperative hypotension but also provides reliable analgesia compared with high-dose local anesthesic[22]. Our meta-analysis has showed that addition of sufentanil to bupivacaine can significantly decrease the probability of breakthrough pain. This meta-analysis may suggest that sufentanil combined with bupivacaine can provide better anesthesia quality than bupivacaine alone, but further study is required to assess side effects such as nausea, and shivering.

Additionally, sufentani and bupivacaine combination also resulted in a shorter time to sensory block and a longer first analgesic request time compared with bupivacaine alone. The faster time of sensory block onset (approximately 1 min) is not likely to be clinically significant for elective cesarean deliveries. However, the faster onset time may be important when initiating anesthesia for emergency cesarean delivery. The high lipid solubility of sufentanil coupled with high affinity forμ-opioid receptors can explain the rapid onset of sensory block[9]. The prolonged sensory block duration can reduce the need for early postoperative analgesia and its possible side effects, and reduce the patient-controlled analgesia cost. Thus, adjuvant opioid sufentanil appears to provide better anesthetic effects including sufficient analgesia, shortened sensory onset, and prolonged first analgesic request time for cesarean delivery surgery.

The main finding of the systematic review by Dahl et al[3] showed that intrathecal morphine prolonged the time to first postoperative analgesic administration, and reducted postoperative pain, whereas fentanyl and sufentanil were not effective to any clinically significant extent. The pooled results of our study showed that sufentanil reduced the breakthrough pain incidence, and prolonged first analgesic request time. There are some differences between Dahl et al’s[3] analysis and ours. Firstly, the local anesthetics in their analysis were different, which included tetracaine, lidocaine and bupivacaine. Secondly, they chose postoperative pain score, and postoperative supplemental analgesic consumption or number of patients needing supplemental analgesics to evaluate the effects of opioids. But we forced on the effects on breakthrough pain in operation, sensory block and motor block.

The addition of sufentanil to bupivacaine does not appear to cause significant adverse neonatal side effects such as the incidences of hypotension and vomiting. However, the incidence of pruritus was higher in the parturients with sufentanil added group. Pooled result indicated that sufentanil did not affect the incidence of maternal nausea; whereas the removal of the trial of Bang et al [14] showed a lower incidence of nausea in the sufentanil added group. Similarly, difference in the incidence of maternal shivering inconclusive in the sensitivity analysis. The clinical significance of the findings about nausea and shivering are unclear and warrants further study.

Maternal hypotension, nausea, and vomiting during spinal anaesthesia during and after cesarean delivery remain common complications[23]. One previous meta-analysis showed that low-dose bupivacaine in spinal anaesthesia could get a lower these common maternal side-effects [24]. The present study suggested that the addition of sufentanil to bupivacaine did not change the risk of maternal arterial hypotension and vomiting.

Pruritus is a well-recognized side effect of spinal opioid analgesia[25]. Pruritus has been reported in 30%–60% of patients who receive spinal opioids[26]. In our meta-analysis, the incidence was 33.0% in the opioids added groups, whlie only 3.1% reported in the control group. Currently, mechanism of intrathecal opioid-induced pruritus is complex and pathogenesis is still not clear[27]. Spinal triggering of itching is observed in particular by activation of μ-opioid receptors[28]. Pruritus has a high incidence in pregnant women (60%–100%)[29–31], and is dose dependent[32, 33]. The increased incidence of pruritus in pregnant women may be due to an interaction of estrogen and opioid receptors[34, 35]. Pruritus invoked by lipid-soluble opioids such as fentanyl and sufentanil is of shorter duration, and the use of the minimum effective dose and addition of local anesthetics seems to decrease the prevalence and the severity of itching[27].

Strict inclusion and exclusion criteria were kept in the present study. The individual influence of included trials were assessed using the risk of bias tool. Most of the trials had high quality, and relevant results were sensitive and stable when data were pooled. Furthermore, comprehensive analysis using Begg’s funnel plots and Egger’s test suggested that the probability of publication bias was small in the present study. Nevertheless, our meta-analysis had a number of limitations. Only sufentanil with the local anesthetic bupivacine was examined. Thus, it is not acceptable that the reported effects of these drugs extend to other opioids and local anesthetics. Additionally, doses varied for both drugs, disabled the recommendation of optimal dose of sufentanil or bupivacaine for clinical application. Not all the included studies systematically listed the data to be examined. For example, motor block and the incidence of shivering were examined in only a limited number of trials. This may result in considerable heterogeneity in our meta-analysis. We attempted to analyze heterogeneity using Different methods were used to analyze the heterogeneity, but any obvious factors that may have contributed to heterogeneity were still not identified. Finally, because of data limitations, pain scores, analgesic requirement, and umbilical cord blood gas outcomes were not analyzed. Only sensory block onset and first analgesic request time, motor block duration and anesthesia quality were selected to evaluate the block effect.

In conclusion, the addition of sufentanil to bupivacaine for spinal anesthesia in patients during cesarean delivery is superior to bupivacaine alone. The significant benefits identified in this meta-analysis include decreased breakthrough pain incidence, shorter sensory block onset time, and longer first analgesic request time. However, increased the incidence of pruritus was the side effect with sufentanil added. Definitive conclusions cannot be drawn regarding the incidence of maternal nausea, shivering and motor block duration. While there are clear clinical advantages to adding sufentanil to bupivacaine for spinal anesthesia, further study is required to determine whether differences exist in the side effect profile of spinal anesthesia with and without sufentanil.

## Supporting Information

S1 Text PRISMA 2009 Checklist. (DOC)
