# Supplementary material for: Sufentanil and Bupivacaine Combination versus Bupivacaine Alone for Spinal Anesthesia during Cesarean Delivery: A Meta-Analysis of Randomized Trials
Source: PLoS One. 2016 Mar 31;11(3):e0152605. doi: 10.1371/journal.pone.0152605 (PMC4816294; doi:10.1371/journal.pone.0152605)
Supplement: S1 Text — (DOC) [file pone.0152605.s001.doc]

| **Section/topic** | **#** | **Checklist item** | **Reported on page #** |
| --- | --- | --- | --- |
| **TITLE** | | |  |
| Title | 1 | Sufentanil and bupivacaine combination versus bupivacaine alone for spinal anesthesia during cesarean delivery: A meta-analysis of randomized trials | 1 |
| **ABSTRACT** | | |  |
| Structured summary | 2 | Objective: The addition of lipophilic opioids to local anesthetics for spinal anesthesia has become a widely used strategy for cesarean anesthesia. A meta-analysis to quantify the benefits and risks of combining sufentanil with bupivacaine for patients undergoing cesarean delivery was conducted.  Methods: A comprehensive literature search without language or date limitation was performed to identify clinical trials that compared the addition of sufentanil to bupivacaine with bupivacaine alone for spinal anesthesia in healthy parturients choosing cesarean delivery. The Q and I2 tests were used to assess heterogeneity of the data. Data from each trial were combined using relative ratios (RRs) for dichotomous data or weighted mean differences (WMDs) for continuous data and corresponding 95% confidence intervals (95% CIs) for each trial. Sensitivity analysis was conducted by removing one study a time to assess the quality and consistency of the results. Begg’s funnel plots and Egger’s linear regression test were used to detect any publication bias.  Results: This study included 9 trials containing 578 patients in the final meta-analysis. Sufentanil addition provided a better analgesia quality with less breakthrough pain during surgery than bupivacaine alone (RR = 0.10, 95% CI 0.06 to 0.18, P < 0.001). Sensory block onset time was shorter and duration was longer in sufentanil added group compared with the bupivacaine-alone group (WMD = −1.0 min, 95% CI −1.5 to −0.58, P < 0.001 and WMD = 133 min, 95% CI 75 to 213, P < 192, respectively). There was no significant difference in the risk of hypotension and vomiting between these two groups. But pruritus was more frequentely reported in the group with sufentanil added (RR = 7.63, 95% CI 3.85 to 15.12, P < 0.001).  Conclusion: Bupivacaine and sufentanil combination is superior to that of bupivacaine alone for spinal anesthesia for cesarean delivery in analgesia quality. Women receiving the combined two drugs had less breakthrough pain, shorter sensory block onset time, and longer sensory block duration. However, the addition of sufentanil to bupivacaine increased the incidence of pruritus**.** | 2-3 |
| **INTRODUCTION** | | |  |
| Rationale | 3 | It was suggested adding opioids to local anesthetic agents for spinal anesthesia might improve anesthesia quality and prolongs the duration of action. Intrathecal administration of opioids is commonly used for cesarean delivery. However, the benefits and risks of this practice with opioids added remain to be fully examined and confirmed. Sufentanil, a lipophilic opioid, was the most frequent drug used in conjunction with the local anesthesitic bupivacaine for cesarean delivery. The aim of this study was to review the analgesic efficacy and side effects of the addition of sufentanil to bupivacaine for spinal anesthesia in healthy parturients undergoing cesarean delivery by a meta-analysis. | 4 |
| Objectives | 4 | The aim of this study was to review the analgesic efficacy and side effects of the addition of sufentanil to bupivacaine for spinal anesthesia in healthy parturients undergoing cesarean delivery by a meta-analysis. | 4 |
| **METHODS** | | |  |
| Protocol and registration | 5 | No protocol and registration for this meta analysis. |  |
| Eligibility criteria | 6 | We included published randomized controlled clinical trials that compared the addition of sufentanil to bupivacaine with identical dose bupivacaine alone used for spinal anesthesia in healthy parturients undergoing scheduled cesarean delivery. | 5 |
| Information sources | 7 | Full articles reporting randomized controlled trials that compared the addition of sufentanil to bupivacaine with bupivacaine alone for cesarean delivery were searched. High-sensitivity and low-specificity search principles were used in MEDLINE, Embase, Cochrane Central Register of Controlled Trials (CENTRAL), and Web of Science without any language or date limitation.. The keywords “cesarean delivery”, “sufentanil”, “spinal anesthesia”, “randomized controlled trial”, and their alternative words were combined by the Boolean meanings of “AND” (for “cesarean delivery”, “sufentanil”, “spinal anesthesia”, “randomized controlled trial”) and “OR” (among alternative words). We also searched the reference lists of relevant articles or textbooks to find other potential studies. The last electronic search was performed in August 2015. | 5 |
| Search | 8 | PUBMED:  ((((((((((((((((((("Cesarean section"[Title/Abstract])) OR ("Cesarean sections"[Title/Abstract])) OR ("Caesarean Section"[Title/Abstract])) OR ("Caesarean Sections"[Title/Abstract])) OR ("Cesarean delivery"[Title/Abstract])) OR ("Cesarean deliveries"[Title/Abstract])) OR ("Caesarean delivery"[Title/Abstract])) OR ("Caesarean deliveries"[Title/Abstract])) OR ("Abdominal Delivery"[Title/Abstract])) OR ("Abdominal Deliveries"[Title/Abstract])) OR ("Postcesarean Section"[Title/Abstract])) OR ("Postcesarean Sections"[Title/Abstract])) OR ("Cesarean Section"[Mesh]))))) AND ((((((((("Spinal Anesthesia"[Title/Abstract])) OR ("Spinal Anesthesias"[Title/Abstract])) OR ("Anesthesia, Spinal"[Mesh])) OR (subarachnoid[Title/Abstract])) OR ("subarachnoid block")) OR (intrathecal[Title/Abstract])) OR ("Injections, Spinal"[Mesh])))) AND (((((((sufentanil[Title/Abstract])) OR (Sulfentanyl[Title/Abstract])) OR (Sulfentanil[Title/Abstract])) OR (sufenta[Title/Abstract])) OR ("Sufentanil"[Mesh])))) AND (((((random*[Title/Abstract])) OR ("Randomized Controlled Trial"[Title/Abstract])) OR ("Randomized Controlled Trial"[Publication Type] OR "Controlled Clinical Trial"[Publication Type]))) | 5 |
| Study selection | 9 | The published papers were reviewed independently by two medical doctors (Zhang C and Hu J). Duplicate studies were excluded redundance from, and then titles, abstracts, and full texts were screened to select the trials that matched the inclusion criteria. | 6 |
| Data collection process | 10 | The inclusion of articles was reviewed independently by two authors. We first excluded duplicate studies, and then screened titles, abstracts, and full text to select the trials that matched the inclusion criteria. | 6 |
| Data items | 11 | Two authors doctotors (Wang R and Wang Y) independently extracted all the relevant information from each included study. Another two doctors checked the consistencey of the extracted data. All doctors involved in data extraction had more than 5 years aneshesiology experience. For each included trial, the following data were collected: the name of the first author, publication year, number of patients, anesthetic dose, the incidence of breakthrough pain requiring supplementary systemic analgesia or conversion to general anesthesia, sensory block onset time (interval from end of anesthetic injection to loss of pain sensitivity to pinprick at predefined dermatome level), sensory block duration (interval from end of anesthetic injection to time of postdelivery complaint of pain that required analgesia treatment), motor block onset time and duration (assessed by modified Bromage scale or Bromage scale), neonatal Apgar scores, and incidence of intraoperative maternal side effects such as hypotension (defined as percent decrease in systolic blood pressure below its baseline value or below an absoluate lower limit, and the definitions were differed among trials), nausea, vomiting, pruritus, and shivering. | 6-7 |
| Risk of bias in individual studies | 12 | We evaluated quality of included trials using the Cochrane Collaboration’s tool for assessing risk of bias in randomized trials. There are seven items to assess random sequence generation, allocation concealment, blinding of participants and personnel, blinding of outcome assessment, incomplete outcome data, selective reporting, and other bias using high, low or unclear risk of bias. | 6 |
| Summary measures | 13 | We plotted relative ratios (RR) (for dichotomous data) or standardized mean differences (SMD) (for continuous data), with corresponding 95% confidence intervals (95% CI) for each trial. | 7 |
| Synthesis of results | 14 | Every analysis was assessed for statistical heterogeneity using the Q-test and the I^2^ test. P <0.10 was considered significant .If P>0.10, I^2^<50%, a fixed effects model was used to analyses. Otherwise, a random effects model was used. | 7 |

Page 1 of 2

| Risk of bias across studies | 15 | Begger's funnel plots and Egger's linear regression test were used to detect the publication bias. We recognized the selective reporting by comparison the integrity of the data, and it also can be recognized by risk bias tools | 7 |
| --- | --- | --- | --- |
| Additional analyses | 16 | To identify sources of heterogeneity, we conducted subgroup analysis and metaa-regression. Sensitivity analysis was conducted by removing each study individually to assess the quality and consistency of the results. Begg’s funnel plots and Egger’s linear regression test were used to detect any publication bias. | 7 |
| **RESULTS** | | |  |
| Study selection | 17 | A total of 405 potentially relevant articles from our search of the literature were identified. After excluding 396 articles, a total of 9 articles covering 9 trials published between 1992 and 2012. | 9 |
| Study characteristics | 18 | A total of 9 articles covering 9 trials published between 1992 and 2012 with 578 patients included (363 received sufentanil in addition to bupivacaine). A summary of the included studies is shown in Table 1.  Five trials examined 1 dose of the drug sufentanil[8-12], and the rest 4 examined more than 1 dose (2 trials examined 2 doses[13,14], 2 trials examined 3 doses[15,16]). Trials that tested more than 1 dose were combined to create a single pair-wise comparison as previously mentioned.   | Year | First author | Bupivacaine Dose (mg) | Sufentanil Dose (ug) | Number of Study/Control | | --- | --- | --- | --- | --- | | | 1997 | Dahlgren[14] | 12.5 | 2.5/5 | 40/20 | | 1998 | Ngiam[10] | 7.5 | 10 | 20/17 | | 2003 | Braga[16] | 12.5 | 2.5/5/7.5 | 60/20 | | 2006 | Demiraran[15] | 12.5 | 1.5/2.5/5.0 | 75/25 | | 2010 | Vyas[12] | 11 | 5 | 30/30 | | 2010 | Veena[11] | 12 | 10 | 20/20 | | 2011 | Lee[9] | near 10 | 2.5 | 24/24 | | 2012 | Braga[8] | 10 | 5 | 24/24 | | 2012 | Bang[13] | near 10 | 2.5/5 | 70/35 | | 9 |
| Risk of bias within studies | 19 | | Table 2：Quality of included trials assessed by Risk bias tools | | | | | | | | | | --- | --- | --- | --- | --- | --- | --- | --- | --- | | Year | First author | Random sequence generation | Allocation concealment | Blinding of participants and personnel | Blinding of outcome assessment | Incomplete outcome data | Selective reporting | Other bias | | 1997 | Dahlgren[14] | Unclear | Low | Low | Low | Low | Low | Unclear | | 1998 | Ngiam[10] | Unclear | Low | Low | Low | Low | Low | Unclear | | 2003 | Braga[16] | Low | Low | Low | Low | Low | Low | Unclear | | 2006 | Demiraran[15] | Low | Unclear | Unclear | Unclear | Unclear | Unclear | Unclear | | 2010 | Vyas[12] | Unclear | Low | Low | Low | Low | Low | Unclear | | 2010 | Veena[11] | Low | Low | Unclear | Unclear | Unclear | Low | Unclear | | 2011 | Lee[9] | Unclear | Unclear | Unclear | Unclear | Low | Low | Unclear | | 2012 | Braga[8] | Low | Low | Unclear | Unclear | Unclear | Unclear | Unclear | | 2012 | Bang[13] | Unclear | Unclear | High | Unclear | Unclear | Low | Unclear | | Table2 |
| Results of individual studies | 20 | Anesthesia quality  Six trials [8-11,13,15] were pooled using a fixed effect model since no heterogeneity was observed (I2 < 0.1%, P = 0.73) when their anesthesia quality were examined. Bupivacaine and sufentanil combination significantly reduced the incidence of breakthrough pain during surgery compared with bupivacaine alone(RR = 0.10, 95% CI 0.06 to 0.18 P < 0.001, Figure 2). Breakthrough pain occurred in 10 of 138 patients in sufentanil group, and in 75 of 103 in bupivacaine-alone group. The result was stable when sensitivity analysis was conducted by removing 1 trial at a time from the pooled result (RRmin = 0.09, 95% CImin 0.04 to 0.18; RRmax = 0.12, 95% CImax 0.07 to 0.21, Figure 3). The Begg’s funnel plots (P = 1.00) and Egger’s linear regression test (P = 0.76) indicated the probability of publication bias was low (Figure 4).  Sensory block and motor block  Sensory block onset time was examined in 4 trials[8,10,12,14]. No heterogeneity was observed according to the I2 and Q tests (I2 = 0.0%, P = 0.81), and therefore, the fixed effects model was selected. Pooled result suggested that sufentanil added to bupivacaine shortened sensory block onset time compared with bupivacaine alone (WMD = -1.04 min, 95% CI -1.50 to -0.58 min; P < 0.001, Figure 5). Removal of individual trials did not significantly alter the result.  Sensory block duration was examined in 7 trials[8,10-14,16]. The possibility of heterogeneity was high (P < 0.001, I² = 98.0%); therefore, subgroup analysis and meta-regression were performed to identify the sources of heterogeneity. Not any source of the heterogeneity was observed, thererfore, a random effect model was selected to pool these results, which demonstrated that the addition of sufentanil prolonged sensory block duration compared with bupivacaine alone (WMD = 133 min, 95% CI 75 to 192, P < 0.001, Figure 6). Again these result was stable when sensitivity analysis that involved removing one trial once from the pooled result was conducted.  Motor block onset time was only examined in 1 trial[14]. Motor block duration was tested in 2 trials[8,14]. A significant heterogeneity was existed in motor block duration according to the I2 and Q tests (P = 0.003, I² = 89.0%). Although subgroup analysis and meta-regression were performed, no source of the heterogeneity was found significant, and the results were pooled using a random effect model. The pooled results suggested that adding sufentanil to bupivacaine did not affect motor block duration compared with bupivacaine alone (WMD = 29 min, 95% CI -19 to 76, P = 0.24, Figure 7). However, when the trial of Dahlgren et al[14] was removed from the pooled trials, the 95% CI was all greater than zero.  Neonatal data  The healthy states of the neonates were evaluated by neonatal Apgar scores 1 and 5 min after delivery. The count of Apgar scores which were lower than 7 after delivery were examined in 6 trials[8,9,13-16]. All the 1-min and 5-min Apgar scores were above 7 in the included trials.  Maternal side effects  Maternal side effects including hypotension [8-11,13,15], nausea [9-13,15,16], vomiting [9,11-16], pruritus [9-17], and shivering[9,11,13] were compared between sufentanil added and bupivacaine alone groups. There were no significant differences in the incidence of hypotension, and vomiting. However, the CIs were wide and heterogeneity was significant. Sufentanil addition significantly increased the incidence of pruritus (Figure 8-12). Sufentanil additiondid not affect the incidence of nausea; however, when the trial of Bang et al [13] was removed from the analysis, the incidence of nausea was significantly lower in the sufentanil combined with bupivacaine group (RR = 0.58, 95% CI 0.40 to 0.85). The pooled results showed that the incidence of shivering was lower in thesufentanil added group, but this conclusion did not stand true when the trial of Lee et al [9] was removed from the analysis (RR = 0.52, 95% CI 0.28 to 0.94). Further evidences maybe required to reach a clear conclusion about the effects on the incidence of nausea and shivering of sufentanil added or bupivacaine alone.  Additional analysis  A significant heterogeneity was identified in the analyses of sensory block duration, motor block duration, and incidence of nausea (I2 > 50%, all P < 0.10). However, the sources of the heterogeneity was not found after having performed subgroup analysis and meta-regression using data sources.  Publication bias was assessed by Begg’s funnel plots and Egger’s linear regression test. All Begg’s funnel plots showed basic symmetry, and Egger’s linear regression test suggested that the probability of publication bias was low (P > 0.05). | 9-13 |
| Synthesis of results | 21 | Anesthesia quality  Six trials [8-11,13,15] were pooled using a fixed effect model since no heterogeneity was observed (I2 < 0.1%, P = 0.73) when their anesthesia quality were examined. Bupivacaine and sufentanil combination significantly reduced the incidence of breakthrough pain during surgery compared with bupivacaine alone(RR = 0.10, 95% CI 0.06 to 0.18 P < 0.001, Figure 2). Breakthrough pain occurred in 10 of 138 patients in sufentanil group, and in 75 of 103 in bupivacaine-alone group. The result was stable when sensitivity analysis was conducted by removing 1 trial at a time from the pooled result (RRmin = 0.09, 95% CImin 0.04 to 0.18; RRmax = 0.12, 95% CImax 0.07 to 0.21, Figure 3). The Begg’s funnel plots (P = 1.00) and Egger’s linear regression test (P = 0.76) indicated the probability of publication bias was low (Figure 4).  Sensory block and motor block  Sensory block onset time was examined in 4 trials[8,10,12,14]. No heterogeneity was observed according to the I2 and Q tests (I2 = 0.0%, P = 0.81), and therefore, the fixed effects model was selected. Pooled result suggested that sufentanil added to bupivacaine shortened sensory block onset time compared with bupivacaine alone (WMD = -1.04 min, 95% CI -1.50 to -0.58 min; P < 0.001, Figure 5). Removal of individual trials did not significantly alter the result.  Sensory block duration was examined in 7 trials[8,10-14,16]. The possibility of heterogeneity was high (P < 0.001, I² = 98.0%); therefore, subgroup analysis and meta-regression were performed to identify the sources of heterogeneity. Not any source of the heterogeneity was observed, thererfore, a random effect model was selected to pool these results, which demonstrated that the addition of sufentanil prolonged sensory block duration compared with bupivacaine alone (WMD = 133 min, 95% CI 75 to 192, P < 0.001, Figure 6). Again these result was stable when sensitivity analysis that involved removing one trial once from the pooled result was conducted.  Motor block onset time was only examined in 1 trial[14]. Motor block duration was tested in 2 trials[8,14]. A significant heterogeneity was existed in motor block duration according to the I2 and Q tests (P = 0.003, I² = 89.0%). Although subgroup analysis and meta-regression were performed, no source of the heterogeneity was found significant, and the results were pooled using a random effect model. The pooled results suggested that adding sufentanil to bupivacaine did not affect motor block duration compared with bupivacaine alone (WMD = 29 min, 95% CI -19 to 76, P = 0.24, Figure 7). However, when the trial of Dahlgren et al[14] was removed from the pooled trials, the 95% CI was all greater than zero.  Neonatal data  The healthy states of the neonates were evaluated by neonatal Apgar scores 1 and 5 min after delivery. The count of Apgar scores which were lower than 7 after delivery were examined in 6 trials[8,9,13-16]. All the 1-min and 5-min Apgar scores were above 7 in the included trials.  Maternal side effects  Maternal side effects including hypotension [8-11,13,15], nausea [9-13,15,16], vomiting [9,11-16], pruritus [9-17], and shivering[9,11,13] were compared between sufentanil added and bupivacaine alone groups. There were no significant differences in the incidence of hypotension, and vomiting. However, the CIs were wide and heterogeneity was significant. Sufentanil addition significantly increased the incidence of pruritus (Figure 8-12). Sufentanil additiondid not affect the incidence of nausea; however, when the trial of Bang et al [13] was removed from the analysis, the incidence of nausea was significantly lower in the sufentanil combined with bupivacaine group (RR = 0.58, 95% CI 0.40 to 0.85). The pooled results showed that the incidence of shivering was lower in thesufentanil added group, but this conclusion did not stand true when the trial of Lee et al [9] was removed from the analysis (RR = 0.52, 95% CI 0.28 to 0.94). Further evidences maybe required to reach a clear conclusion about the effects on the incidence of nausea and shivering of sufentanil added or bupivacaine alone. | 9-12 |
| Risk of bias across studies | 22 | Publication bias was assessed by Begg's funnel plots and Egger's linear regression test. The results we found suggest all the comparisons did not have statistically significant publication bias. At the same time, the Begg's funnel plots were basic symmetry.  We did not find any obvious bias in the included studies. | 11 |
| Additional analysis | 23 | A significant heterogeneity was identified in the analyses of sensory block duration, motor block duration, and incidence of nausea (I2 > 50%, all P < 0.10). However, the sources of the heterogeneity was not found after having performed subgroup analysis and meta-regression using data sources.  Publication bias was assessed by Begg’s funnel plots and Egger’s linear regression test. All Begg’s funnel plots showed basic symmetry, and Egger’s linear regression test suggested that the probability of publication bias was low (P > 0.05). | 13 |
| **DISCUSSION** | | |  |
| Summary of evidence | 24 | Spinal anesthesia is routinely used for cesarean delivery because of its ease of control, fast onset, effective nerve block, low failure rate, and low systemic toxicity[18,19]. In addition, it can decrease the risk of airway complications[19,20]. However, using local anesthetics alone for spinal anesthesia may be inadequate[18]. It has been suggested that the addition of various opioids to local anesthetics may improve intra- and post-operative analgesic effects and reduce side effects[13,14,16,19] . A previous meta-analysis had examined the effects of adding opioids for many minor surgeries but cesarean delivery was not included[2]. The present meta-analysis comparing the addition of sufentanil to bupivacaine with bupivacaine alone for spinal anesthesia found that the addition of sufentanil to bupivacaine provided significant benefits for spinal anesthesia in healthy parturients during cesarean delivery as compared to bupivacaine alone.  A clinically important effect of adding lipid-soluble sufentanil to bupivacaine is the significantly lower incidence of breakthrough pain. Studies suggested that spinal anesthesia with high-dose local anesthetic (e.g., bupivacaine 12-15 mg) provided effective analgesia, but a high incidence of hypotension was complicated[10,13]. Although spinal anesthesia by low-dose local anesthetic without opioid is with a low incidence of hypotension, but also a high probabilility of failed surgical anesthesia was concerned. Low-dose bupivacaine combined with opioid not only reduces the incidence of intraoperative hypotension but also provides reliable analgesia compared with high-dose local anesthesic[21]. Our meta-analysis has showed that addition of sufentanil to bupivacaine can significantly decrease the probability of breakthrough pain. This meta-analysis may suggest that sufentanil combined with bupivacaine can provide better anesthesia quality than bupivacaine alone, but further study is required to assess side effects such as nausea, and shivering.  Additionally, sufentani and bupivacaine combination also resulted in a shorter time to sensory block and a longer sensory block duration compared with bupivacaine alone. The faster time of sensory block onset (approximately 1 min) is not likely to be clinically significant for elective cesarean deliveries. However, the faster onset time may be important when initiating anesthesia for emergency cesarean delivery. The high lipid solubility of sufentanil coupled with high affinity forμ-opioid receptors can explain the rapid onset of sensory block[8]. The prolonged sensory block duration can reduce the need for early postoperative analgesia and its possible side effects, and reduce the patient-controlled analgesia cost. Thus, adjuvant opioid sufentanil appears to provide better anesthetic effects including sufficient analgesia, shortened sensory onset, and prolonged sensory block duration for cesarean delivery surgery.  The addition of sufentanil to bupivacaine does not appear to cause significant adverse neonatal side effects such as the incidences of hypotension and vomiting. However, the incidence of pruritus was higher in the parturients with sufentanil added group. Pooled result indicated that sufentanil did not affect the incidence of maternal nausea; whereas the removal of the trial of Bang et al [13] showed a lower incidence of nausea in the sufentanil added group. Similarly, difference in the incidence of maternal shivering inconclusive in the sensitivity analysis. The clinical significance of the findings about nausea and shivering are unclear and warrants further study.  Maternal hypotension, nausea, and vomiting during spinal anaesthesia during and after cesarean delivery remain common complications[22]. One previous meta-analysis showed that low-dose bupivacaine in spinal anaesthesia could get a lower these common maternal side-effects [23]. The present study suggested that the addition of sufentanil to bupivacaine did not change the risk of maternal arterial hypotension and vomiting.  Pruritus is a well-recognized side effect of spinal opioid analgesia[24]. Pruritus has been reported in 30%–60% of patients who receive spinal opioids[25]. In our meta-analysis, the incidence was 33.0% in the opioids added groups, whlie only 3.1% reported in the control group. Currently, mechanism of intrathecal opioid-induced pruritus is complex and pathogenesis is still not clear[26]. Spinal triggering of itching is observed in particular by activation of μ-opioid receptors[27]. Pruritus has a high incidence in pregnant women (60%–100%)[28-30], and is dose dependent[31,32]. The increased incidence of pruritus in pregnant women may be due to an interaction of estrogen and opioid receptors[33,34]. Pruritus invoked by lipid-soluble opioids such as fentanyl and sufentanil is of shorter duration, and the use of the minimum effective dose and addition of local anesthetics seems to decrease the prevalence and the severity of itching[26]. Trials showed that intravenous administration of ondansetron and propofol are both effective in the treatment of pruritus-induced by intrathecal use of sufentanil in obstetric patients[35]. | 14-18 |
| Limitations | 25 | Strict inclusion and exclusion criteria were kept in the present study. The individual influence of included trials were assessed using the risk of bias tool. Most of the trials had high quality, and relevant results were sensitive and stable when data were pooled. Furthermore, comprehensive analysis using Begg’s funnel plots and Egger’s test suggested that the probability of publication bias was small in the present study. Nevertheless, our meta-analysis had a number of limitations. Only sufentanil with the local anesthetic bupivacine was examined. Thus, it is not acceptable that the reported effects of these drugs extend to other opioids and local anesthetics. Additionally, doses varied for both drugs, disabled the recommendation of optimal dose of sufentanil or bupivacaine for clinical application. Not all the included studies systematically listed the data to be examined. For example, motor block and the incidence of shivering were examined in only a limited number of trials. This may result in considerable heterogeneity in our meta-analysis. We attempted to analyze heterogeneity using Different methods were used to analyze the heterogeneity, but any obvious factors that may have contributed to heterogeneity were still not identified. Finally, because of data limitations, pain scores, analgesic requirement, and umbilical cord blood gas outcomes were not analyzed. Only sensory block onset and duration, motor block duration and anesthesia quality were selected to evaluate the block effect. | 17-18 |
| Conclusions | 26 | In conclusion, the addition of sufentanil to bupivacaine for spinal anesthesia in patients during cesarean delivery is superior to bupivacaine alone. The significant benefits identified in this meta-analysis include decreased breakthrough pain incidence, shorter sensory block onset time, and longer sensory duration. However, increased the incidence of pruritus was the side effect with sufentanil added. Definitive conclusions cannot be drawn regarding the incidence of maternal nausea, shivering and motor block duration. While there are clear clinical advantages to adding sufentanil to bupivacaine for spinal anesthesia, further study is required to determine whether differences exist in the side effect profile of spinal anesthesia with and without sufentanil. | 18-19 |
| **FUNDING** | | |  |
| Funding | 27 | Xiangya Clinic Trial Fund(2013L13)  National Natural Science Foundation of China(81400921) |  |
